# Neurogenic factor-induced Langerhans cell activation in diabetic mice with mechanical allodynia

**DOI:** 10.1186/1742-2094-10-64

**Published:** 2013-05-14

**Authors:** Jacqueline R Dauch, Diane E Bender, Lucía A Luna-Wong, Wilson Hsieh, Brandon M Yanik, Zachary A Kelly, Hsinlin T Cheng

**Affiliations:** 1 Department of Neurology, University of Michigan Medical Center, Ann Arbor, MI, USA; 2 Department of Neurology, University of Michigan, 109 Zina Pitcher Place, 5015 Biomedical Science Research Building, Ann Arbor, MI 48109-2200, USA

**Keywords:** Langerhans cells, Painful diabetic neuropathy, p38, Type 2 diabetes, Tumor necrosis factor-α, Mechanical allodynia, db/db mouse

## Abstract

**Background:**

Langerhans cells (LCs) are antigen-presenting dendritic cells located in the skin. It has been reported that LC activation is associated with painful diabetic neuropathy (PDN); however, the mechanism of LC activation is still unclear.

**Methods:**

The db/db mouse, a rodent model of PDN, was used to study the roles of LCs in the development of PDN in type 2 diabetes. Hind foot pads from db/db and control db/+ mice from 5 to 24 weeks of age (encompassing the period of mechanical allodynia development and its abatement) were collected and processed for immunohistochemistry studies. LCs were identified with immunohistochemistry using an antibody against CD207 (Langerin). The intraepidermal nerve fibers and subepidermal nerve plexus were identified by immunohistochemistry of protein gene product 9.5 (PGP 9.5) and tropomyosin-receptor kinase (Trk) A, the high affinity nerve growth factor receptor.

**Results:**

CD207-positive LCs increased in the db/db mouse during the period of mechanical allodynia, from 8 to 10 weeks of age, in both the epidermis and subepidermal plexus. At 16 weeks of age, when mechanical allodynia diminishes, LC populations were reduced in the epidermis and subepidermal plexus. Epidermal LCs (ELCs) were positive for Trk A. Subepidermal LCs (SLCs) were positive for CD68, suggesting that they are immature LCs. Additionally, these SLCs were positive for the receptor of advanced glycation end products (RAGE) and were in direct contact with TNF-α-positive nerve fibers in the subepidermal nerve plexus during the period of mechanical allodynia. Intrathecal administration of SB203580, a p38 kinase inhibitor, significantly reduced mechanical allodynia, TNF-α expression in the subepidermal plexus, and increased both ELC and SLC populations during the period of mechanical allodynia.

**Conclusions:**

Our data support the hypothesis that increased LC populations in PDN are activated by p38-dependent neurogenic factors and may be involved in the pathogenesis of PDN.

## Background

Diabetes is a prevalent illness worldwide and it has been estimated that 4.4% (330 million) of the world population will be affected by 2030 [1]. The majority of diabetic patients (>90%) have type 2 diabetes, which is tightly linked to obesity and insulin insensitivity. Diabetic neuropathy (DN) is the most common diabetic complication that affects at least 50% of patients with either type 1 or type 2 diabetes [2,3]. At least 40% to 50% of patients with DN develop painful diabetic neuropathy (PDN), which presents as abnormal sensory symptoms including allodynia (increased sensitivity to innocuous stimuli), hyperalgesia (exaggerated response to noxious stimuli), and/or spontaneous pain [4,5]. Although there are multiple presentations of PDN in diabetic patients, the most common symptoms of PDN result from a length-dependent polyneuropathy that is associated with damage of peripheral nerve endings in the skin [6]. Despite increased animal and human research over the last two decades, our understanding of the distal nerve pathology that contributes to the development of PDN remains limited. Current PDN treatments, such as anticonvulsants, antidepressants and α2δ calcium channel antagonists, do not target the pathomechanisms that are specific for PDN. Therefore, only less than 30% of patients obtain satisfactory pain relief [7].

Accumulating evidence suggests that inflammatory mechanisms are essential for the development of complications in type 2 diabetes [8-12]. These mechanisms involve increased levels of proinflammatory cytokines and chemokines, as well as activation of inflammatory cells including macrophages, T/B lymphocytes, and dendritic cells [8,13-15]. However, it is unclear if these inflammatory phenomena are involved in the pathogenesis of PDN from type 2 diabetes. Here, we use db/db mice, a model for type 2 diabetes, to study the role of skin inflammation that could contribute to peripheral nerve damage and pain during the period of mechanical allodynia in db/db mice from 6 to 12 weeks of age [16]. In this period, we detected nerve growth factor (NGF)/p38-dependent upregulation of inflammatory markers, including TNF-α, nitric oxide synthases and cyclooxygenase (COX) 2, in db/db mice [17]. Our findings suggested that there is increased inflammation in the peripheral nerves in db/db mice during the period of mechanical allodynia.

Langerhans cells (LCs) are dendritic cells in the skin which regulate local immune reactions [18]. LCs are derived from monocytes that migrate to the dermis from bone marrow during development [19,20]. In conditions of skin infection or inflammation, CD68-positive LC precursors in the dermis proliferate and differentiate into CD207-positive mature LCs in the epidermis, where they serve as antigen-presenting cells to uptake pathogens and activate T cells [19,21]. It has been reported that increased LC numbers in the skin correlate with PDN in both animal and human studies [22,23].

In the current study, we hypothesize that LCs are activated in the skin of db/db mice in response to increased levels of neurogenic inflammatory markers in order to mediate mechanical allodynia. We examined the epidermal CD207-positive/CD68-negative LCs and subepidermal CD207-positive/CD68-positive LCs in the hind foot pads of db/db and control db/+ mice. Our results provide evidence that inhibition of NGF/p38 signaling in peripheral nerves could prevent LC-mediated immune activation in skin, and serve as a potential treatment for PDN.

## Methods

### Animals

Male C57BLKS db/db mice were purchased from The Jackson Laboratory (stock number 000662, Bar Harbor, ME, USA). The homozygous (*Lepr*^*db*^/*Lepr*^*db*^, or db/db) mouse was used as a model of type 2 diabetes, while the heterozygous mouse (*Lepr*^*db*^/+, or db/+) served as the nondiabetic control. Analyses and procedures were performed in compliance with protocols established by the Animal Models of Diabetic Complications Consortium (AMDCC), and approved by the University Committee on Use and Care of Animals (UCUCA) at the University of Michigan. All possible efforts were made to minimize the animals’ suffering and the number of animals used.

### Immunohistochemistry

Hind foot pads were collected, immersed for 6 to 8 hours at 4°C in Zamboni’s fixative (2% paraformaldehyde, 0.2% picric acid in 0.1 M phosphate buffer), rinsed in 30% sucrose in PBS solution overnight, cryoembedded in mounting media (optimal cutting temperature (OCT) compound), and sectioned at 30 μm thick before being processed for immunohistochemistry.

Tissue sections were processed for CD68, CD207, protein gene product 9.5 (PGP 9.5), tropomyosin-receptor kinase (Trk) A, the receptor for advanced glycation end products (RAGE), and TNF-α immunohistochemistry. Sections were incubated at room temperature for 16 to 24 hours with primary antibodies: CD68 (1:200, LifeSpan BioSciences, Seattle, WA, USA), CD207 (1:1000, Abcam Biochemicals, Cambridge, MA, USA), PGP 9.5 (1:2000, Millipore, Billerica, MA, USA), Trk A (1:500, R&D Systems, Minneapolis, MN, USA), RAGE (1:500, Santa Cruz Biotechnology, Santa Cruz, CA, USA), and TNF-α (1:500, Abcam Biochemicals). Sections were then rinsed three times in PBS and incubated with secondary antiserum conjugated with different fluorophores (Alexa Fluor 488, 594, or 647, Invitrogen, Carlsbad, CA, USA). Sections were rinsed and mounted with ProLong Gold Antifade Reagent (Invitrogen). In order to confirm that there were no nonspecific immunoreactions, additional sections were incubated with primary or secondary antisera alone. Fluorescent images were collected on an Olympus FluoView 500 (Center Valley, PA, USA) confocal microscope using a 40 × 1.2 oil immersion objective at a resolution of 1024 × 1024 pixels. The optical section thickness was 0.5 μm. Approximately 40 images per stack were flattened using the MetaMorph (version 6.14, Molecular Devices, Sunnyvale, CA, USA) arithmetic option. Six sections were measured for each foot pad. Cell density data were presented as the mean number of cells per linear millimeter of epidermis from a total of 12 sections per animal.

### SB203580 treatment

An osmotic minipump (ALZET model 1007D, DURECT Corporation, Cupertino, CA, USA) was used for continuous intrathecal infusion into the lumbar spinal cord region. The 100 μl volume minipump is designed with a 0.51 μl/h infusion rate. The minipumps were filled with artificial cerebrospinal fluid (CSF) that contained 10% dimethyl sulfoxide (DMSO) with or without SB203580 (1 mg/ml, EMD Chemicals, Gibbstown, NJ, USA), a p38 kinase inhibitor. The minipumps were implanted into the dorsal subcutaneous space between the shoulder blades of each mouse at 7 weeks of age under sterile conditions. A caudally directed polyethylene cannula (Becton, Dickinson and Company, Sparks, MD, USA) was threaded subcutaneously at the level of the L5 spinal process by removing the L5 spinal process and inserting the tip of the cannula into the subarachnoid space. The intrathecal infusion lasted for 1 week, after which hind foot pads were collected for immunohistochemistry.

### Data presentation and statistical analyses

All data are presented as group means ± SEM. The data between db/+ and db/db mice of the same age were analyzed using the Mann–Whitney test. Statistical comparisons between different age groups were made by a one-way ANOVA test followed by a post-hoc Tukey’s multiple comparison test. A *P* value of less than 0.05 was considered statistically significant.

## Results

### Subepidermal and epidermal CD207-positive LCs in foot pad skin of db/db mice increase during the period of mechanical allodynia

**Figure 1:**
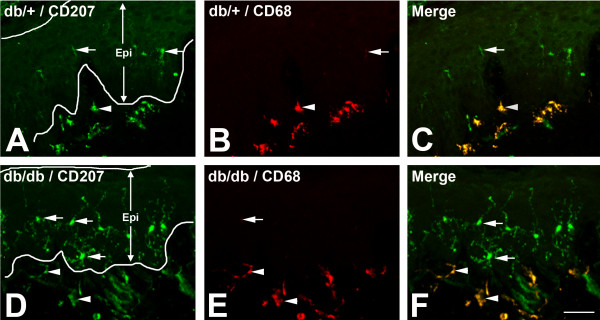
**Increased quantity of CD207-positive LCs in the epidermis and subepidermal plexus were detected in db/db mice during the period of mechanical allodynia.** Representative double immunofluorescent images of CD207 (green: **A**,**C**,**D**,**F**) and CD68 (red: **B**,**C**,**E**,**F**) immunohistochemistry on hind foot pads from db/+ (**A**,**B**,**C**) and db/db mice (**D**,**E**,**F**) at 8 weeks of age. Increased numbers of CD207-positive cells in the epidermal Langerhans cells (ELCs, arrows) and subepidermal Langerhans cells (SLCs, arrowheads) were detected in db/db mice (compare **A** and **D**). In contrast, trace CD68 immunoreactivity was observed in ELCs of db/db mice (**E**, arrows). SLCs were mostly positive for CD68 (**B**,**E**, arrowheads). Bar = 50 μm, n = 8. ELC, epidermal Langerhans cell; Epi, epidermis; LC, Langerhans cell; SLC, subepidermal Langerhans cell.

To study the LC population in skin during the period of mechanical allodynia, we performed CD207 immunohistochemistry on foot pad skin of control db/+ and diabetic db/db mice from 5 to 24 weeks of age. Figure 1 demonstrates representative confocal images of CD207 immunohistochemistry from foot pads of db/+ (Figure 1A,B,C) and db/db mice (Figure 1D,E,F) at 8 weeks of age. CD207 immunohistochemistry detected LCs in the epidermal (ELCs, Figure 1, arrows) and the subepidermal plexus (SLCs, Figure 1, arrowheads). Increased numbers of both ELCs and SLCs were detected in db/db mice compared to db/+ mice (Figure 1). In addition, CD68 immunohistochemistry, which labels immature LCs, was performed concomitantly using double immunofluorescence. Strong CD68 immunoreactivity was detected in CD207-positive SLCs of both db/+ and db/db mice (Figure 1B,E, arrowheads), with only minimal signal detected in CD207-positive ELCs (Figure 1B,E, arrows).

### The LC population increases in skin at 8 to 10 weeks of age and declines at 16 weeks of age

**Figure 2:**
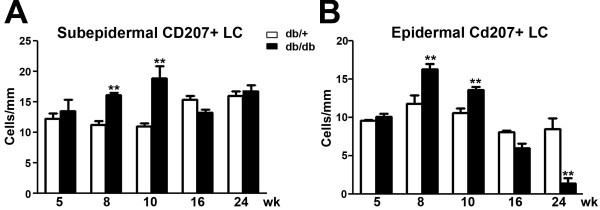
**Quantification of CD207-positive LC densities in the subepidermal plexus and epidermis of db/+ and db/db mice during the time period of mechanical allodynia.** (**A**) Increased densities (cells/mm) of CD207-positive cells were detected in the subepidermal plexus at 8 and 10 weeks of age in db/db mice compared to db/+ mice of the same age. (**B**) Increased densities of CD207-positive cells were detected in the epidermal space at 8 and 10 weeks of age in db/db mice compared to db/+ mice of the same age. At 16 weeks, both subepidermal and epidermal LC densities of db/db mice were reduced to the control levels of db/+ mice. In addition, the epidermal CD207-positive cell densities were reduced at 24 weeks in db/db mice compared to db/+ mice. **P <0.01, n = 8. LC, Langerhans cell.

Quantitative cell density studies at 5 to 24 weeks of age (Figure 2) demonstrated that increased densities of CD207-positive SLCs (Figure 2A) and ELCs (Figure 2B) were detected at 8 to 10 weeks, during the period of mechanical allodynia. The proliferation of both SLCs and ELCs subsided after 16 weeks when SLC density returned to the control level. In contrast, the ELC density dropped below the control level at 24 weeks of age.

### ELCs express Trk A

**Figure 3:**
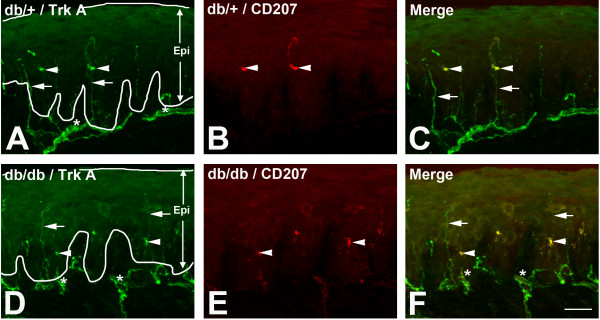
**CD207-positive LCs express Trk A immunoreactivity.** Representative images of Trk A/CD207 double immunohistochemistry of hind foot pads from db/+ (**A**,**B**,**C**) and db/db (**D**,**E**,**F**) mice at 8 weeks of age. Trk A immunoreactivity was detected in both IENFs (**A**,**D**, arrows) and nerve fibers in the subepidermal nerve plexus (**A**,**D**, asterisks) in both db/+ and db/db mice. In addition, CD207-positive LCs were positive for Trk A (**B**,**C**,**E**,**F**, arrowheads). Most CD207-positive cells also expressed Trk A immunoreactivity in both db/+ (**B**, arrowheads) and db/db mice (**E**, arrowheads). Bar = 50 μm, n = 8. Epi, epidermis; IENF, intraepidermal nerve fiber; LC, Langerhans cell; Trk, tropomyosin-receptor kinase.

We next investigated mechanisms for the increase in the ELC population in db/db mice. LC proliferation could be triggered by growth factors, cytokines, and chemokines [20]. First, we tested if NGF could be a mitogen present to mediate LC expansion in db/db mice. We performed CD207/Trk A double immunohistochemistry on hind foot pad sections of db/+ (Figure 3A,B,C) and db/db (Figure 3D,E,F) mice. As demonstrated in Figure 3, most LCs were positive for Trk A in both db/+ and db/db mice.

### SLCs aggregate along the subepidermal plexus

**Figure 4:**
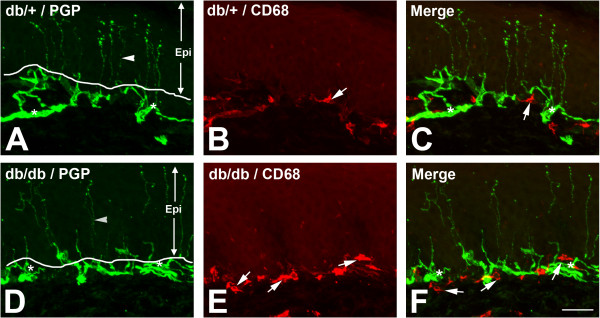
**Increased aggregation of CD68-positive cells in the subepidermal plexus of db/db mice.** Representative images of PGP 9.5/CD68 double immunohistochemistry of hind foot pads from db/+ (**A**,**B**,**C**) and db/db (**D**,**E**,**F**) mice at 8 weeks of age. PGP 9.5 immunohistochemistry demonstrates IENFs (**A**,**D**, arrowheads,) and nerve fibers located in the subepidermal nerve plexus (**A**,**C**,**D**,**F**, asterisks). Increased quantities of subepidermal CD68-positive cells (**B**,**C**,**E**,**F**, arrows) were detected in the subepidermal plexus (**A**,**C**,**D**,**F**, asterisks). Bar = 50 μm, n = 8. Epi, epidermis; IENF, intraepidermal nerve fiber; PGP, protein gene product.

**Figure 5:**
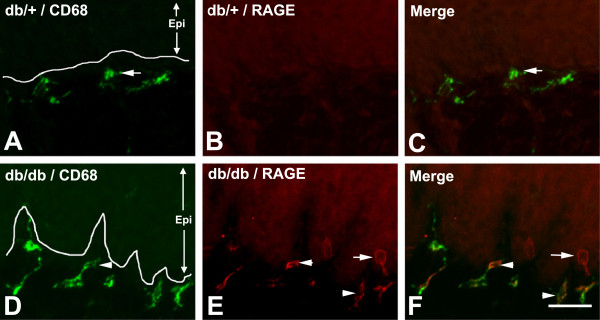
**Subepidermal CD68-positive cells of db/db mice are positive for RAGE.** Representative images of CD68/RAGE double immunohistochemistry of hind foot pads from db/+ (**A**,**B**,**C**) and db/db (**D**,**E**,**F**) mice at 8 weeks of age. RAGE immunoreactivity was only detected in db/db mice (**E**, arrowheads) but not db/+ mice (**B**). Most RAGE-positive cells are positive for CD68 (**E**, arrowheads) with some exceptions (**E**, arrow). Bar = 50 μm, n = 8. Epi = epidermis; RAGE, receptor for advanced glycation end products.

To examine the cell densities of SLCs within the subepidermal plexus, we performed double immunofluorescent studies for CD68 and PGP 9.5, a pan-neuronal marker. PGP 9.5 immunohistochemistry identified intraepidermal nerve fibers (IENFs, arrowheads) and nerve fibers in the subepidermal plexus (Figure 4A,D, asterisks). Compared to db/+ mice, increased numbers of SLCs (Figure 4B,C,E,F, arrows) were detected along the subepidermal plexus (compare Figure 4B and E). Most of these SLCs were also positive for RAGE in db/db mice (Figure 5E,F, arrowheads), but not in db/+ mice (Figure 5B,C). In addition, some CD68-negative cells express RAGE in the subepidermal plexus of db/db mice (Figure 5E,F, arrows).

### Increased SLCs are associated with enhanced TNF-α expression in the subepidermal plexus

**Figure 6:**
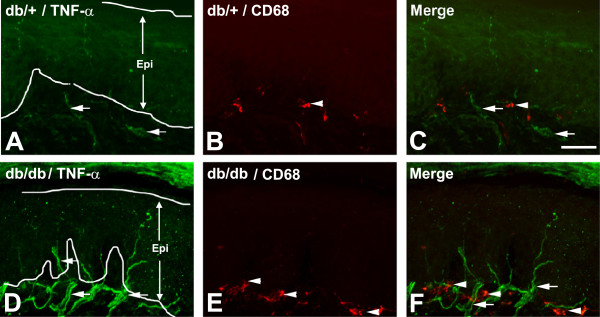
**Increased TNF-α expression in the subepidermal plexus of db/db mice.** Representative images of TNF-α/CD68 double immunohistochemistry of hind foot pads from db/+ (**A**,**B**,**C**) and db/db (**D**,**E**,**F**) mice at 8 weeks of age. Increased TNF-α immunoreactivity was detected in the subepidermal plexus (**A**,**D**, arrows), along with increased quantities of subepidermal CD68-positive cells (**B**,**E**, arrowheads). Bar = 50 μm, n = 8. Epi, epidermis.

We then investigated the mechanisms that could contribute to the aggregation of LCs in the skin of db/db mice. We speculated that TNF-α could be a chemotactic factor that attracts SLCs to the subepidermal plexus. We performed TNF-α immunohistochemistry. As demonstrated in Figure 6, TNF-α expression was mostly detected in the subepidermal plexus and IENFs of db/+ and db/db mice (Figure 6A,C,D,F, arrows). Increased TNF-α immunoreactivity was detected in db/db mice compared to db/+ mice (compare Figure 6A and D). Most of the CD68-positive SLCs were in contact with TNF-α-positive nerve fibers in db/db mice (Figure 6F). In addition, some of the CD68-positive cells also expressed TNF-α immunoreactivity in db/db mice (Figure 6F, arrowheads).

### SB203580 inhibits increased TNF-α expression and proliferation of CD68-positive SLCs and CD207-positive ELCs in db/db mice

**Figure 7:**
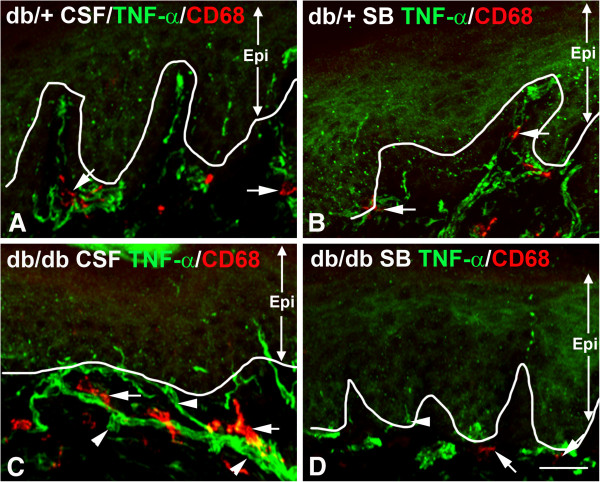
**Intrathecal SB203580 treatment reduces TNF-α expression and the quantity of CD68-positive cells in the subepidermal plexus of db/db mice.** Representative images of TNF-α/CD68 double immunohistochemistry of hind foot pads from db/+ (**A**,**B**) and db/db (**C**,**D**) mice at 8 weeks of age after 1 week of intrathecal artificial cerebrospinal fluid (CSF) or SB203580 (SB) treatment. Compared to the CSF-treated db/db mice, SB203580 treatment reduced the expression of TNF-α in the subepidermal plexus (compare **C** and **D**, arrowheads) in the db/db treated mice. In contrast, SB203580 treatment did not affect TNF-α (arrowheads) or CD68 (arrows) expression in the subepidermal plexus of db/+ mice (compare **A** and **B**). Bar = 50 μm, n = 8. CSF, cerebrospinal fluid; Epi, epidermis.

**Figure 8:**
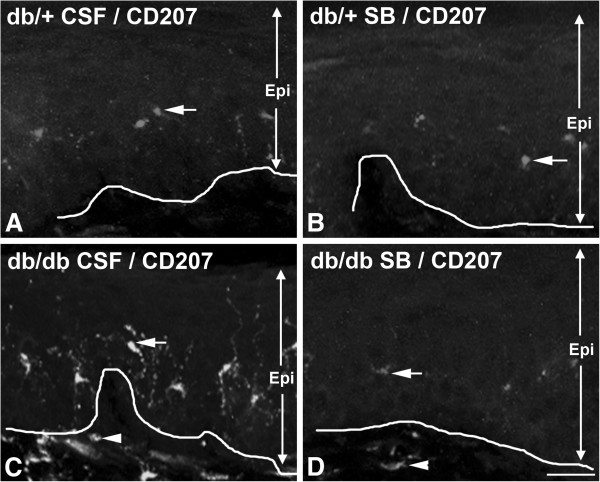
**Intrathecal SB203580 treatment reduces the quantity of CD207-positive cells in the epidermis of db/db mice.** Representative images of CD207 immunohistochemistry of hind foot pads from db/+ (**A**,**B**) and db/db (**C**,**D**) mice at 8 weeks of age after 1 week of intrathecal artificial cerebrospinal fluid (CSF) or SB203580 (SB) treatment. Compared to the CSF-treated db/db mice, SB203580 treatment reduced the quantity of CD207-positive cells in the epidermis (compare **C** and **D**, arrows) of the treated db/db mice. In contrast, SB203580 treatment had no effect on the quantity of CD207-positive cells in the epidermis of db/+ mice (compare **A** and **B**). Bar = 50 μm, n = 8. CSF, cerebrospinal fluid; Epi, epidermis.

**Figure 9:**
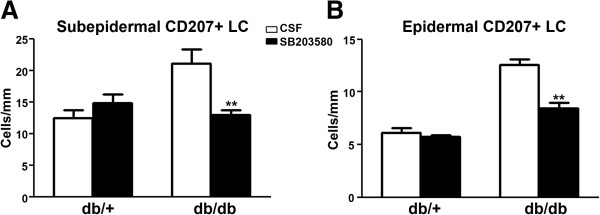
**Intrathecal SB203580 treatment reduces LC density in the subepidermal plexus and epidermis of db/db mice.** (**A**) SB203580 treatment reduced SLC density in db/db mice, but had no effect on db/+ mice. (**B**) SB203580 treatment reduced ELC density in db/db mice, but had no effect on db/+ mice. **P <0.01, n = 8. CSF, cerebrospinal fluid; ELC, epidermal Langerhans cell; LC, Langerhans cell; SLC, subepidermal Langerhans cell.

Previously, we established that NGF/p38 signaling mediates the development of mechanical allodynia in db/db mice [17]. Using the same approach, we examined the hypothesis that p38 signaling in dorsal root ganglia (DRG) mediates increased LC aggregation in skin and TNF-α expression in the subepidermal plexus of db/db mice during the period of mechanical allodynia. As described previously, we administered control artificial CSF or SB203580, a p38 inhibitor, via minipumps intrathecally for 1 week starting at 7 weeks of age [17]. Foot pads were collected at the end of the treatment at 8 weeks of age, and processed for CD68 and TNF-α immunohistochemistry. As demonstrated in Figure 7, SB203580 significantly reduced the number of CD68-positive SLCs (compare Figure 7C and D, arrows) and TNF-α immunoreactivity (compare Figure 7C and D, arrowheads) in db/db mice. In contrast, SB203580 did not affect CD68 and TNF-α immunoreactivity in db/+ mice (compare Figure 7A and B). The effect of SB203580 on the ELC population was studied by CD207 immunohistochemistry (Figure 8). Similar to CD68-positive SLCs, SB203580 reduced the numbers of CD207-positive ELCs in db/db mice (compare Figure 8C and D, arrows), but had no effect in db/+ mice (compare Figure 8A and B, arrows). Quantification of cell density revealed a significant reduction of both SLC and ELC cell densities in db/db mice due to SB203580 treatment (Figure 9).

## Discussion

In the current study, we report the mechanisms of a novel neurogenic skin inflammation that occurs in PDN of type 2 diabetes. We detected increased quantities of LCs in the epidermis and subepidermal plexus in diabetic mice during the period of mechanical allodynia. This inflammatory phenomenon is initiated by p38 signaling in DRG neurons.

Our data suggest increased quantities of ELCs in db/db mice during the period of mechanical allodynia. Increased ELCs have been reported in animal models and patients with DN. Lauria and colleagues reported increased PGP 9.5-positive LCs in the skin of rats treated with streptozotocin [23]. A similar phenomenon has been described in the cornea of patients with DN. Tavakoli and colleagues reported that increased corneal LCs correlates with corneal nerve damage in diabetic patients [24]. The time course of the observed increase in the LC population correlated with the period of mechanical allodynia. Consistent with the current study, Casanova-Molla *et al*. demonstrated increased LC populations in skin biopsies of human diabetic subjects with painful phenotypes [22]. However, the observed increase in LC populations of painful conditions is not specific to PDN, as it has also been reported in models of other painful neuropathies [25] and peripheral nerve injury [26,27].

There are two potential mechanisms for increased LC populations in foot pad skin of diabetic mice. First, LCs could proliferate in the epidermis in response to diabetic conditions [20]. Second, there could be increased recruitment of dermal immature LCs to the epidermis. The current findings support that both mechanisms coexist. Our results demonstrate that the expression of Trk A in epidermal LCs support the first mechanism. The Trk A-positive LCs could respond to increased NGF from the nerves at this stage and proliferate. In support of our hypothesis, Noga and colleagues reported that NGF activates Trk A-positive human monocyte-derived dendritic cells to mediate asthma [28]. In addition to direct NGF mitogenic actions on LCs, NGF-dependent factors such as prostaglandins, nitric oxide, substance P, calcitonin gene related peptides, TNF-α, and other cytokines could also contribute to ELC expansion [17,29-31].

The second proposed mechanism is supported by our data that demonstrate increased CD68-positive and CD207-positive cells in the subepidermal plexus during the period of mechanical allodynia. This mechanism occurs during early embryonic development when immature LCs migrate out of the bone marrow and distribute throughout the dermis and the dermal-epidermal junction before they enter the epidermis [32]. Immature LCs, located in the dermis (identified by CD207-positivity) are derived from monocytes that are positive for CD68 [33]. In conditions of dermatitis, similar recruitment of immature LCs and monocytes from bone marrow to the skin has been reported [33,34]. This action is mediated by the CCR2 and CCR6 chemokine receptors, and colony-stimulating factor-1 signaling [19,34].

We additionally present data that demonstrate that CD68-positive and CD207-positive SLCs express RAGE in db/db mice. This result suggests that diabetes-induced RAGE expression might contribute to the increased SLC population in db/db mice. RAGE expression is important for the homing of maturing dendritic cells to lymph nodes where they physically interact and activate T lymphocytes [35]. Thus, it is possible that RAGE mediates the maturation and migration of SLCs to the epidermis where they can activate skin immunity, evidence which further supports the second mechanism.

Additionally, we demonstrate the upregulation of TNF-α in the subepidermal plexus of diabetic mice. TNF-α is a cytokine that mediates both acute and chronic inflammation [36]. Furthermore, increased TNF-α actions cause neuropathic pain in models of nerve injury or neuropathy [17,37]. Previously, we reported that TNF-α is upregulated in DRG neurons in db/db mice during the period of mechanical allodynia by a NGF/p38-dependent mechanism [17]. In addition to PDN, upregulated TNF-α expression was detected in the skin and muscle nerve afferents by enhanced anterograde axonal transport after nerve injury [38,39]. The increased TNF-α level is known to mediate pain by triggering ectopic activity in afferent sensory nerve fibers [40,41]. In addition, regional TNF-α administration also activates LC migration [42], a likely mechanism for increased LC aggregation toward peripheral nerves, as demonstrated in the current study. The current results suggest that p38 is an essential mediator for LC-mediated immunoreaction in PDN of type 2 diabetes. Previously, we reported that intrathecal SB203580 treatment reduced mechanical allodynia [17] and the upregulation of IENF densities in db/db mice [43]. Since SB203580 was administered intrathecally, we can conclude that p38 activation in the nervous system contributes to LC aggregation in the skin. Similar neurogenic inflammation has been reported in other models of neuropathic pain, including nerve injury by complete or partial nerve transection and chronic constriction injury [44]. In the nerve injury model, inflammatory cells such as macrophages, mast cells, dendritic cells, and Schwann cells secrete proinflammatory cytokines, chemokines, growth factors, nitric oxide, and other pain mediators to enhance neuropathic pain [44]. Along with other researchers in the field, we reported that COX2, TNF-α and nitric oxide synthases are upregulated by a NGF/p38-dependent mechanism [17]. However, further studies are necessary to explore other factors that could contribute to the development of PDN in type 2 diabetes.

## Conclusions

In summary, we detected increased LC populations in the epidermis and subepidermal plexus of db/db mice during the period of mechanical allodynia. This phenomenon is triggered by p38-dependent signaling mechanisms that include the upregulation of TNF-α in the subepidermal plexus. Our results support the hypothesis that inhibition of p38 signaling and/or TNF-α actions could be effective treatments for PDN.

## Abbreviations

AMDCC: Animal models of diabetic complications consortium; ANOVA: Analysis of variance; COX: Cyclooxygenase; CSF: Cerebrospinal fluid; DMSO: Dimethyl sulfoxide; DN: Diabetic neuropathy; DRG: Dorsal root ganglia; ELC: Epidermal langerhans cell; IENF: Intraepidermal nerve fiber; LC: Langerhans cell; NGF: Nerve growth factor; OCT: Optimal cutting temperature; PBS: Phosphate buffered saline; PDN: Painful diabetic neuropathy; PGP: Protein gene product; RAGE: Receptor for advanced glycation end products; SEM: Standard error of the mean; SLC: Subepidermal langerhans cell; TNF: Tumor necrosis factor; Trk: Tropomyosin-receptor kinase; UCUCA: University committee on use and care of animals.

## Competing interests

The authors declare that they have no competing interests.

## Authors’ contributions

JD and HC drafted and revised the manuscript, study concept and design, analysis and interpretation of data, acquisition of data, and statistical analysis. DB drafted and revised the manuscript, study concept, and experimental design. LL-W, WH, ZK, and BY acquired the data. All authors read and approved the final version of the manuscript.
